# Amino acid profile in overweight and obese prepubertal children – can simple biochemical tests help in the early prevention of associated comorbidities?

**DOI:** 10.3389/fendo.2023.1274011

**Published:** 2023-10-26

**Authors:** Jolanta Bugajska, Joanna Berska, Małgorzata Wójcik, Krystyna Sztefko

**Affiliations:** ^1^ Department of Clinical Biochemistry, Institute of Pediatrics, Jagiellonian University Medical College, Krakow, Poland; ^2^ Department of Pediatric and Adolescent Endocrinology, Institute of Pediatrics, Jagiellonian University Medical College, Krakow, Poland

**Keywords:** obesity, prepubertal children, amino acids, metabolic dysfunction-associated fatty liver disease, cardiovascular disease, alanine aminotransferase, uric acid

## Abstract

**Background:**

It is accepted that plasma branched-chain amino acids (BCAAs) and aromatic amino acids (AAAs) are closely related to metabolic risk. Arterial hypertension, metabolic syndrome, endothelial dysfunction, inflammation, and metabolic dysfunction-associated fatty liver disease (MAFLD) are frequently seen in obese patients. Many attempts have been made to find biochemical indicators for the early detection of metabolic complications in children. It is not known if different amino acid profiles and BCAA and AA concentrations in overweight and obese children correlate with chemerin, proinflammatory, and simple biochemical markers. Thus, the study aimed to find out the early markers of cardiovascular disease and MAFLD in overweight and obese children.

**Materials and methods:**

The study included 20 overweight and obese children (M/F 12/8; mean age 7.7 ± 2.3 years; BMI 26.8 ± 5.0 kg/m^2^) and 12 non-obese children (control group) (M/F 4/8; mean age 6.5 ± 2.2 years; BMI 14.8 ± 1.5 kg/m^2^). The following plasma amino acids were measured: aspartic acid, glutamic acid, serine, asparagine, glycine, glutamine, taurine, histidine, citrulline, threonine, alanine, arginine, proline, tyrosine, methionine, valine, isoleucine, leucine, phenylalanine, tryptophan, ornithine, and lysine. Chemerin, high-sensitivity C-reactive protein (hs-CRP), interleukin-6 (IL-6), and basic biochemistry parameters were measured.

**Results:**

The mean plasma levels of leucine, isoleucine, valine, phenylalanine, tyrosine, glutamic acid, and alanine were significantly higher in overweight and obese children than in the control group (p<0.03–p<0.0004). Conversely, the mean values of serine, asparagine, glutamine, and citrulline were significantly lower in overweight and obese children than in the control group (p<0.03–p<0.0007). Isoleucine, leucine, valine (BCAAs) tyrosine, and phenylalanine (AAAs) levels showed a positive correlation with uric acid, ALT, hs-CRP, and chemerin (r=0.80–0.36; p<0.05-p<0.00001), but not with IL-6. The mean values of glucose, IL-6, hs-CRP, chemerin, uric acid, and ALT were significantly higher in overweight and obese children than in the control group (p<0.03–p<0.00002). In contrast, the lipid profile did not differ between groups.

**Conclusion:**

An abnormal amino acid profile in overweight and obese pre-pubertal children, accompanied by elevated ALT and UA observed in the studied cohort, may suggest early metabolic disturbances that can potentially lead to metabolic syndrome, or MAFLD, and increased cardiovascular risk.

## Introduction

Obesity constitutes a significant health problem for children and adolescents all over the world. It is associated with the early development of cardiovascular disease and non-alcoholic fatty liver disease (NAFLD), recently redefined as metabolic dysfunction-associated fatty liver disease (MAFLD) (1). The increasing prevalence of obesity in children and adolescents calls for simple biochemical markers useful for quick screening of children at the highest risk for the development of cardiovascular disease. Obesity is closely connected to the pathophysiology of cardiovascular diseases. Adipose tissue produces many adipokines, among them chemerin. It is known that plasma chemerin is increased in patients with coronary artery disease and plays an important role in promoting adipogenesis, preadipocyte differentiation, adipocyte development, and glucose metabolism (2). It has even been hypothesized that chemerin at high concentrations increases the risk of major adverse cardiovascular events (3). In addition, chemerin may be considered a diagnostic biomarker to monitor the development and progression of metabolic dysfunction-associated fatty liver disease in children. There have been significant associations between the circulating levels of chemerin and the presence of MAFLD (4, 5). Plasma concentrations of amino acids (AAs) are often increased in MAFLD (6). Consumption of foods high in fat and protein contributes to the development of obesity. Dietary protein is comprised of more than 20% BCAAs, which are particularly elevated in MAFLD (7). Branched-chain amino acids (BCAAs; valine, isoleucine, and leucine) and aromatic amino acids (AAAs; tyrosine, phenylalanine, and tryptophan) are closely associated with metabolic risk.

The amino acid profile in diabetes may reflect metabolic changes not only in the disease *per se* but also in its complications. Based on the amino acid profile, information on every single amino acid concentration can be used for the prevention and treatment of the patient (8–10). Elevated levels of BCAAs are significantly associated with obesity in children and adolescents and may independently predict insulin resistance in the future (11). In children and adolescents (9-19 years) with severe obesity, elevated concentrations of BCAAs (calculated by adding valine, leucine, and isoleucine levels were observed (12). This indicates that obesity, MAFLD, and other metabolic pathways involved in lipid and glucose metabolism are linked (12). High BCAAs may be useful in the identification of many obesity complications such as insulin resistance, dyslipidemia, and MAFLD, which was nicely summarized in the review paper (13). In addition, the association between daily BCAA intake and increased risk of overweight and insulin resistance was observed in children of mothers with gestational diabetes mellitus (14).

On the other hand, many biochemical indicators are increased in arterial hypertension, metabolic syndrome, endothelial dysfunction, inflammation, and MAFLD, conditions frequently seen in obese patients.

MAFLD is a growing health problem in the pediatric population. Among simple, routinely performed laboratory tests, alanine aminotransferase (ALT) could be considered sufficient to diagnose MAFLD in these children and could predict MAFLD in the future (15). Another simple molecule that is measured routinely, uric acid (UA), is present in high concentrations in patients with metabolic syndrome when the latter is associated with endothelial dysfunction, inflammation, and hypertension. High concentrations of UA may play a key role in cardiovascular diseases (16). Hyperuricemia may influence vascular function by exerting pro-oxidant effects and decreasing nitric oxide bioavailability, followed by the induction of inflammation and endothelial dysfunction, and may promote hypertension and cardiovascular disease (17, 18).

Chronic low-grade inflammation and hyperglycemia, which promote disease development, may be reflected in amino acid alterations. Additionally, chronic low-grade inflammatory states have been hypothesized to contribute to the development of depression in obese individuals (19). It is known that depression has been found to predict coronary heart disease (20). Decreased tryptophan availability to the brain in patients with obesity may play a role in the pathogenesis of inflammation-induced depression. Tyrosine, valine, isoleucine, leucine, and phenylalanine compete with tryptophan for transport across the blood–brain barrier, so they are referred to as competing amino acids (CAAs) (21). The tryptophan:CAA ratio has also been associated with depression (21) and obesity (22, 23).

However, it is not known whether different amino acid profiles and BCAA and AA concentrations in overweight and obese children correlate with chemerin, proinflammatory, and simple biochemical markers. Thus, the aim of the study was to find out the early markers of cardiovascular disease and MAFLD in overweight and obese children.

## Materials and methods

The study included 20 overweight and obese children (study group) (M/F 12/8; mean age 7.7 ± 2.3 years; BMI 26.8 ± 5.0 kg/m^2^) and 12 children without overweight or obesity (control group) (M/F 4/8; mean age 6.5 ± 2.2 years; BMI 14.8 ± 1.5 kg/m^2^). Overweight and obesity were determined using the Polish BMI percentile charts for children aged 3–18 years. Overweight was defined as a BMI above the 85th percentile (>1SD) and obesity as a BMI above the 97th percentile (>2SD) (24). In the study group, six children had a family history of obesity, type 2 diabetes, and hypertension; three children had a family history of obesity and hypertension; six children had a family history of obesity; and five children out of 20 had no family history of obesity, type 2 diabetes, and hypertension. The children in the study group and the control group were healthy, without infections or chronic diseases, nor were they taking any medication. The control group was recruited from patients with suspected endocrine diseases who were finally excluded. Plasma-free amino acids (AAs), chemerin, high-sensitivity C-reactive protein (hs-CRP), interleukin-6 (IL-6), glucose, uric acid (UA), alanine aminotransferase (ALT), aspartate aminotransferase (AST), creatinine, and lipid profile (total cholesterol, low-density lipoprotein cholesterol (LDL-C), high-density lipoprotein cholesterol (HDL-C), triglycerides (TG)) were determined. The study was approved by the Bioethics Committee of the Jagiellonian University (Protocol No. 1072.6120.331.2020). Written informed consent was obtained from all parents before their children were included in the study. The study was carried out in accordance with the Declaration of Helsinki.

### Biochemical analyses

A fasting venous blood sample was drawn from each patient into a lithium heparin tube and into tubes containing separating gel. The blood was centrifuged for 10 min at 1200×g. Plasma samples were kept at −70°C until analysis of amino acids, chemerin, hs-CRP, and IL-6 concentrations. Routine serum biochemistry tests: glucose, UA, ALT, AST, creatinine, total cholesterol, HDL-C, and TG were measured by dry chemistry, and LDL-C was measured by wet chemistry (Vitros 4600, Ortho Clinical Diagnostics Inc., Rochester, NY, USA). Plasma-free amino acid concentrations were measured using a highly selective liquid chromatography-tandem mass spectrometry method (LC-MS/MS, 1260 Infinity II, 6460 QTRAP; Agilent Technologies, Waldbronn, Germany) with a quantitative amino acid analysis kit (Jasem, Istanbul, Turkey). The following plasma AAs were measured: valine, isoleucine, leucine, threonine, methionine, phenylalanine, lysine, tryptophan, glutamine, histidine, arginine, tyrosine, aspartic acid, glutamic acid, serine, asparagine, glycine, taurine, citrulline, alanine, proline, ornithine, 3-methyl-histidine, cystine, and α-aminobutyric acid. Plasma competing amino acids (CAAs) were calculated by summing the concentrations of tyrosine, valine, isoleucine, leucine, and phenylalanine. Additionally, the tryptophan:CAA ratio (tryptophan availability) was calculated. Chemerin, hs-CRP, and IL-6 in plasma were measured using commercially available ELISA kits (R&D, Minneapolis, MN, USA).

### Statistical analysis

Statistical analysis was performed using Statistica version 13 (StatSoft, Kraków, Poland). Data distribution was checked using the Shapiro–Wilk test. Data were presented as mean ± SD or median (interquartile range). Comparisons between the study group and the control groups were made using the t-test for parametric data or the Mann-Whitney U test for non-parametric data. Spearman’s correlation was used to examine the relationships between isoleucine, leucine, valine, tyrosine, phenylalanine, UA, ALT, hs-CRP, and chemerin. For ALT and UA, the ROC (Receiver Operating Characteristic) curves were constructed, and the AUC (Area under the Curve) was computed. The level of significance was set at a p-value of less than 0.05.

## Results

The mean levels of chemerin, hs-CRP, IL-6, UA, ALT, and glucose were significantly higher in overweight and obese children than in the control group (p<0.03 – p<0.00002), whereas AST, creatinine, and lipid profile did not differ between groups (**Table 1**). The mean plasma levels of leucine, isoleucine, valine, phenylalanine, tyrosine, glutamic acid, and alanine were significantly higher in overweight and obese children than in the control group (p<0.03 – p<0.0004) (**Table 2**). Conversely, the mean values of serine, asparagine, glutamine, and citrulline were significantly lower in overweight and obese children than in controls (p<0.03–p<0.0007) (**Table 2**). The mean tryptophan index (tryptophan:CAA) was significantly lower in overweight and obese patients than in controls.

**Table 1:** The mean ± SD or median (interquartile range) values of chemerin, hs-CRP, IL-6, and basic biochemistry in the control group (non-overweight and non-obese) and in the study group (overweight and obese).

	Control group	Study group		p	
	Mean ± SD or Median (Interquartile range)				
Chemerin [pg/ml]	799.3± 98.0	973.7 ± 104.5		**0.00006**	
hs-CRP [ng/ml]	1.26 (0.56-2.11)	18.8 (7.43-52.3)		**0.00001**	
IL-6 [pg/ml]	0.71 (0.58-1.29)	2.07 (1.56-2.87)		**0.0009**	
Uric acid [µmol/l]	221.0 ± 49.5	296.2 ± 63.4		**0.001**	
ALT [U/l]	14.5 (13.0–18.5)	27.0 (17.5–33.0)		**0.001**	
Glucose [mmol/l]	4.3 ± 0.5	4.7 ± 0.4		**0.03**	
AST [U/l]	36.3 ± 6.7	33.9 ± 5.8		0.31	
Creatinine [µmol/l]	38.1 ± 7.4	42.4 ± 8.3		0.14	
Total cholesterol [mmol/l]	4.38 (3.78–4.72)	4.03 (3.61–4.51)		0.45	
HDL cholesterol [mmol/l]	1.35 ± 0.42	1.23 ± 0.22		0.29	
LDL cholesterol [mmol/l]	2.46 (2.11-2.57)	2.13 (1.94–3.00)		0.70	
Triglycerides [mmol/l]	0.72 (0.59–1.16)	0.86 (0.71–1.45)		0.34	

Bold fonts mean statistically significant values.

**Table 2:** The mean ± SD or median (interquartile range) values of amino acids in the control group (non-overweight and non-obese) and the study group (overweight and obese).

Amino acids	Control group		Study group	p
	Mean ± SD or Median (Interquartile range) [µmol/l]			
Valine	189.9± 27.9		257 ± 53.7	**0.0004**
Isoleucine	55.8 ± 8.0		70.9 ± 14.6	**0.003**
Leucine	77.2 ± 13.9		96.2 ± 18.4	**0.004**
Threonine	106.0 ± 18.7		119.3 ± 18.5	0.06
Methionine	23.5 ± 3.9		24.7 ± 3.1	0.36
Phenylalanine	53.1 (47.5–55.9)		61.7 (54.4–73.1)	**0.008**
Lysine	149.3 (136.2–167.7)		170.5 (150,6–183.2)	0.09
Tryptophan	67.9 ± 10.2		73.2 ± 12.4	0.22
Glutamine	691.8 ± 71.5		609.7 ± 79.9	**0.007**
Histidine	75.2 (72.1–78.5)		78.7 (68.1–83.3)	0.98
Arginine	77.2 (71.7–92.1)		56.2 (45.8–88.4)	0.11
Tyrosine	47.5 (38.4–52.2)		63.3 (55.7–78.7)	**0.002**
Aspartic acid	0.80 (0.57–2.58)		1.59 (0.78–3.15)	0.60
Glutamic acid	17.4 (10.4–26.5)		39.0 (28.1–54.1)	**0.003**
Serine	124.4 ± 21.8		107.6 ± 17.5	**0.02**
Asparagine	52.1 ± 6.7		44.5 ± 5.8	**0.002**
Glycine	323.6 (283.6-369.0)		280.3 (232.3-337.6)	0.09
Taurine	49.4 (44.1–61.2)		52.4 (48.0–67.0)	0.32
Citrulline	38.8 ± 6.1		27.5 ± 5.9	**0.0007**
Alanine	286.4 (254.4–361.4)		370.9 (319.8–471.7)	**0.03**
Proline	129.3 (114.9–197.3)		170.9 (119.1–195.1)	0.55
Ornithine	54.2 ± 13.2		60.8 ± 18.6	0.29
3-methyl-histidine	1.54 ± 0.52		2.02 ± 0.40	**0.006**
Cystine	31.6 ± 5.6		38.0 ± 8.1	**0.02**
α-Aminobutyric acid	13.6 ± 4.8		13.8 ± 4.0	0.87
Index				
tryptophan:CAA	0.16 ± 0.03		0.13 ± 0.02	**0.0027**

CAA = Tyrosine + Valine + Isoleucine + Leucine + Phenylalanine.

Bold fonts mean statistically significant values.

Isoleucine, leucine, valine, tyrosine and phenylalanine levels showed a positive correlation with UA, ALT, hs-CRP, and chemerin (r=0.80 – 0.36; p<0.05 - p<0.00001) (**Table 3**; **Supplementary Material Figures 1**, **2**), but not with IL-6 when results from all children were used for calculation.

**Table 3:** Correlation between amino acids (isoleucine, leucine, valine, phenylalanine, tyrosine) and ALT, uric acid, hs-CRP, and chemerin in all children.

	ALT [U/l]		uric acid [µmol/l]		chemerin [pg/ml]		hs-CRP [ng/ml]	
	r	p	r	p	r	p	r	p
isoleucine	0.5602	0.0009	0.5937	0.0003	0.4753	0.0060	0.5446	0.0013
leucine	0.6315	0.0001	0.4210	0.0164	0.4337	0.0132	0.5534	0.0010
valine	0.7950	<0.00001	0.3622	0.0416	0.5221	0.0022	0.6295	0.0001
phenylalanine	0.3615	0.0420	0.5421	0.0014	0.6433	0.00007	0.6568	0.00004
tyrosine	0.6518	0.00005	0.3934	0.0286	0.6469	0.00006	0.5452	0.0013

r, correlation coefficient; p, p-value.

ROC curves were constructed for ALT and UA. The AUC for ALT (0.853; confidence interval 0.727 – 0.979) and for UA (0.849; confidence interval 0.719 – 0.979) were computed. ROC curves showed the discriminatory capacity between overweight and obese children and healthy children. The cutoff point for ALT was 24 U/l and for UA was 270.5 µmol/l.

## Discussion

Detection of early metabolic changes in children before puberty should be a high priority. Early modification of dietary habits, lifestyle, and/or pharmacological intervention in overweight and obese children should protect them from more severe complications. As this study has shown, the amino acid profile in overweight and obese children differs from the one seen in non-overweight and non-obese children. It is known that amino acids, besides being necessary for protein synthesis, play an important role in obesity-related diseases such as cardiovascular disease and liver disease.

The relationship between insulin and BCAAs is well known. Initially, chronic elevation of leucine and isoleucine may contribute to hyperinsulinism and, consequently, may lead to beta cell failure. Two mechanisms have been suggested to explain the relationship between BCAAs and insulin resistance/type 2 diabetes mellitus. First, an increased level of BCAAs may lead to an increased number of toxic compounds in BCAA metabolism in the mitochondria, causing toxic damage to pancreatic beta cells and consequently impairing insulin secretion. Second, an excess of BCAAs may activate the mTORC1 complex, which may promote insulin resistance (13). The association between high levels of circulating BCAAs in the blood and also the association between daily intake of BCAAs and increased risk of overweight and insulin resistance in patients has been noted (14). Insulin concentration was not measured because the relationship between insulin and BCAAs was not considered in the present study. We also did not evaluate daily BCAA intake by using a validated food frequency questionnaire. In our previous study, we investigated BCAA intake before and after meal consumption in adults. The fasting and postprandial levels of BCAAs were higher in the study group (patients with cholecystolithiasis) than in the control group, regardless of whether they were measured in the fasting state or postprandial state (25). We are aware that the lack on data of amino acid intake is a limitation of the study, but to look for the link between BCAAs and simple biochemical parameters, we did not need a dietary questionnaire. What we needed was to find out whether high BCAAs correlate with simple biochemical parameters. Measuring the biochemistry panel of hundreds of patients every day in every laboratory makes it easy to look at ALT activity and uric acid concentration. If both are even slightly elevated, then an in-depth study of dietary habits in children should be performed.

Da Silva et al. (26) found an association between abdominal obesity and homocysteine and cysteine concentrations in prepubertal children. Homocysteine and cysteine may be early and independent predictors of cardiovascular risk (26). Our study has shown that cystine (cystine is the main form of extracellular cysteine) was higher in overweight and obese prepubertal children than in the control group. Elevated plasma BCAAs are known to be associated with cardiovascular disease risk, but the mechanisms by which BCAAs affect cardiac function remain poorly understood (27). In the present study, the mean fasting levels of BCAAs were higher in the study group than in the controls. Additionally, significant positive correlations were found between isoleucine, leucine, valine, UA, ALT, hs-CRP, and chemerin. In the previous study we demonstrated the association between isoleucine, leucine, valine, phenylalanine, tyrosine, and BMI in girls with an obesity diagnosis (28). The same results were obtained by He et al. (29), who demonstrated that branched-chain amino acids and aromatic amino acids were positively correlated with BMI. Our findings confirm the important interaction between obesity and metabolic health. Amino acid metabolism is altered in obese children even before pubertal onset.

Tryptophan catabolism is altered in the metabolic syndrome. Mallmann et al. (30) found a positive correlation between UA and an increased conversion of tryptophan to kynurenine (kynurenine:tryptophan ratio) in adult patients. These factors, in combination with inflammation, may collectively determine CVD risk (30). Depression and mood disorders may contribute to coronary heart disease (20). While fasting plasma tryptophan did not differ significantly between the studied groups, we found that the mean tryptophan index (tryptophan:CAA ratio) was significantly lower in patients with obesity than in controls (p = 0.0027). This may indicate decreased tryptophan availability to the brain and the possibility of the development of inflammation-induced depression. Inflammation biomarkers, endothelial dysfunction, and parameters associated with metabolic syndrome are elevated in obese prepubertal children and correlate with UA levels (31). We found significant differences in inflammatory markers (CRP, IL-6) between the overweight and obese prepubertal children and the controls. Obese adolescents with hyperuricemia demonstrate significant elevations in markers of metabolic syndrome, such as serum glucose and triglycerides (32). In our group of prepubertal overweight and obese children, dyslipidemia was not present. In addition, a prospective observational study showed that elevated serum UA levels independently predicted an increased risk of incident MAFLD (33).

Chemerin has been proposed as a novel biomarker for the early diagnosis and prognosis of cardiovascular disease (3). As shown in previous studies, it may be associated with early vascular pathology and the risk of hypertension in obese children (34). Ba et al. (35) found significantly higher chemerin levels in obese children and adolescents than in the control group. We obtained similar results. The mean value of chemerin was significantly higher in obese children than in the control group, and significant positive correlations were observed between BCAAs, AAAs, and chemerin. High levels of chemerin contribute to the chronic low-grade inflammation associated with obesity and to obesity-related conditions such as cardiovascular disease. Cosentino et al. (36) showed significant correlations between BCAAs, AAAs, and hs-CRP in obese youth. BCAAs and AAAs may link adiposity-related dysfunction to enhanced CVD risk and may be biomarkers of CVD in obese adolescents (36).

In this study, the mean values of glutamic acid and ALT were significantly higher in overweight and obese children compared to controls. This may be due to increased transamination. There are two isoforms of human ALT, namely ALT1 and ALT2. ALT1 plays a significant role in the kidney, liver, and heart, whereas ALT2 may play a significant role, particularly in tissues such as muscle, fat, and the brain. Adipose tissue is not highly active in gluconeogenesis. ALT2 in fat tissue may participate in the generation of pyruvate and thus glyceroneogenesis, contributing to the homeostasis of fatty acid metabolism and their storage (37). Liver transaminases play a role in the regulation of systemic metabolic function. ALT is a pyridoxal enzyme that catalyzes the reversible transamination between alanine and 2-oxoglutarate to form pyruvate and glutamate. Glutamate is the anion of glutamic acid. Gaggini et al. (6) showed significantly higher concentrations of glutamate and ALT in adult patients with MAFLD (both non-obese and obese) compared to healthy controls. Additionally, they noted higher concentrations of alanine, valine, isoleucine, leucine, tyrosine, phenylalanine, and lysine and lower concentrations of glycine in obese patients with MAFLD compared to the controls (6). We found significantly higher mean concentrations of alanine, valine, isoleucine, leucine, tyrosine, and phenylalanine in overweight and obese children as compared to the controls. Also, the mean concentration of lysine was higher and glycine was lower in the study group than in the control group, but these differences were not significant. The observed amino acid profile in overweight and obese prepubertal children is the same as in obese patients with MAFLD. It is hypothesized that in patients with MAFLD, decreased plasma concentrations of glycine and serine are due to increased use of serine and glycine in the synthesis of glutathione, while glutamate is increased due to increased transamination (6).

Metabolic disturbances have been observed in young prepubertal overweight and obese children (38). Screening for MAFLD in overweight and obese children is recommended by pediatric, endocrinology, and gastroenterology societies. Schwimmer et al. (39) estimated the diagnostic performance of ALT in overweight and obese children over 10 years of age identified as having MAFLD based on primary care screening. According to the cited authors, ALT >80 U/l (two times the upper limit of normal) would increase the specificity of ALT for the diagnosis of MAFLD. However, many children with MAFLD would be missed (39). Our study groups were under 10 years of age. In our opinion, the cut-off point for ALT >80 U/l is much too high because we are looking for small changes in the reference range that can be detected within it. It is known that the biological intraindividual variability of ALT is high in the general population, but quite low in children, so that small increases in ALT activity are easily identifiable.

The results of the present study support the idea of re-evaluating the normative values of ALT and UA. In this study, both mean serum ALT and UA concentrations were within the normal reference range (10 – 35 U/l and 120 – 320 µmol/l, respectively) in both the study and control groups. The currently used upper limit for ALT does not clearly discriminate between the presence and absence of liver disease. Lin et al. (40) proposed the upper limit of ALT (23 U/L for boys and 18 U/L for girls) to screen metabolic dysfunction-associated fatty liver disease in obese children in the Taiwanese population. According to the recommendations of the North American Society of Pediatric Gastroenterology, Hepatology, and Nutrition (NASPGHAN), the best screening test for MAFLD in children is the measurement of ALT activity, taking into account normal values of <22 U/l for girls and <26 U/l for boys. Individual laboratory upper limits of normal are not recommended (41). Screening for MAFLD should be considered between 9 and 11 years of age in obese children and overweight children if they have additional risk factors (41). In the present study, a value of 24 U/l is proposed as the upper limit of the reference range for ALT, and a value of 270.5 µmol/l is proposed as the upper limit of the reference range for UA in prepubertal children (between 5 and 9 years of age for obese and overweight children). Further research is required to reevaluate and validate the ALT normative values using a larger cohort.

Limitations of the study: 1) The research was performed on a small number of children; 2) Amino acid intake data were not available.

Strengths of the study: 1) The research was performed on carefully selected, homogeneous prepubertal children aged 5-9 years from one geographical region, without potential selection bias. Thus, the results of the study can be generalized to the Caucasian pediatric population, but only to prepubertal children; 2) The idea of the study linking amino acid profiles with simple biochemical measurements is original and no such approach can be found in the literature.

In conclusion, the abnormal amino acid profile in overweight and obese prepubertal children associated with elevated ALT and UA observed in the studied cohort may suggest early metabolic disturbances that may potentially lead to metabolic syndrome, or MAFLD, and increased cardiovascular risk.

## Data availability statement

The raw data supporting the conclusions of this article will be made available by the authors, without undue reservation.

## Ethics statement

The studies involving humans were approved by Jagiellonian University Bioethics Committee (Protocol No. 1072.6120.331.2020). The studies were conducted in accordance with the local legislation and institutional requirements. Written informed consent for participation in this study was provided by the participants’ legal guardians/next of kin.

## Author contributions

JBu: Conceptualization, Data curation, Formal Analysis, Funding acquisition, Investigation, Methodology, Resources, Software, Validation, Visualization, Writing – original draft, Writing – review & editing. JBe: Data curation, Methodology, Resources, Writing – review & editing. MW: Conceptualization, Formal Analysis, Investigation, Resources, Writing – review & editing. KS: Project administration, Supervision, Validation, Writing – review & editing.

## Supplementary material

SUPPLEMENTARY FIGURE 1 Correlation between **(A)** Isoleucine, **(B)** leucine, **(C)** valine, **(D)** phenylalanine, **(E)** tyrosine and ALT, and uric acid (all children).

SUPPLEMENTARY FIGURE 2 Correlation between **(A)** Isoleucine, **(B)** leucine, **(C)** valine, **(D)** phenylalanine, **(E)** tyrosine and hs-CRP, and chemerin (all children).
