# Supplementary figures and images for: Amino acid profile in overweight and obese prepubertal children – can simple biochemical tests help in the early prevention of associated comorbidities?
Source: Front Endocrinol (Lausanne). 2023 Oct 26;14:1274011. doi: 10.3389/fendo.2023.1274011 (PMC10641253; doi:10.3389/fendo.2023.1274011)

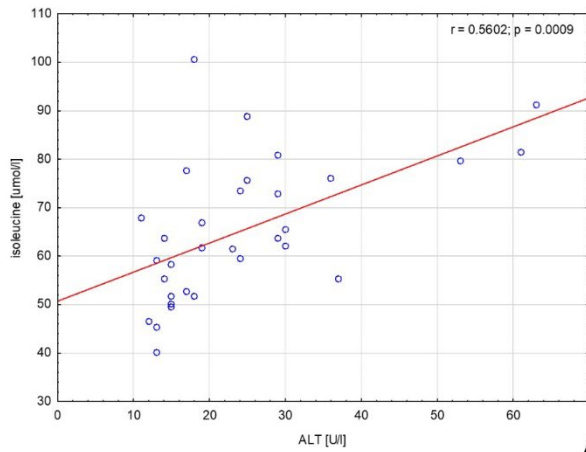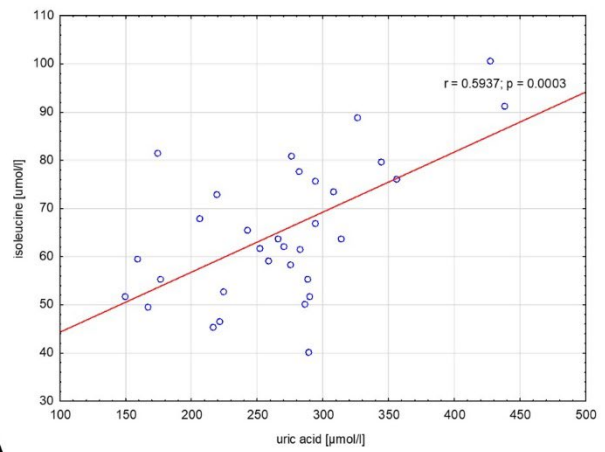

A

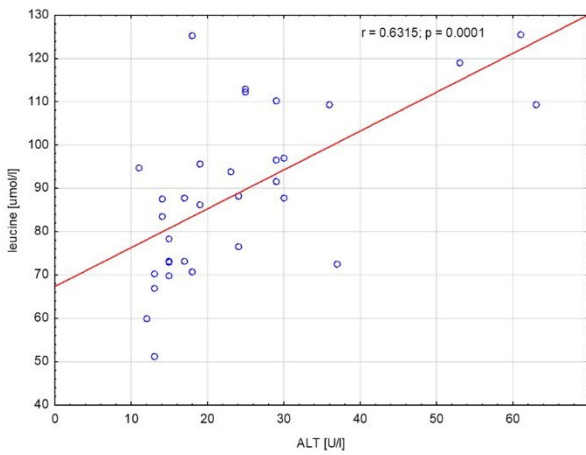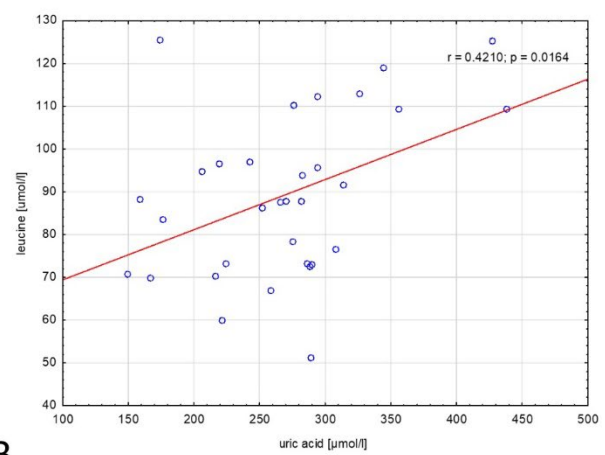

B

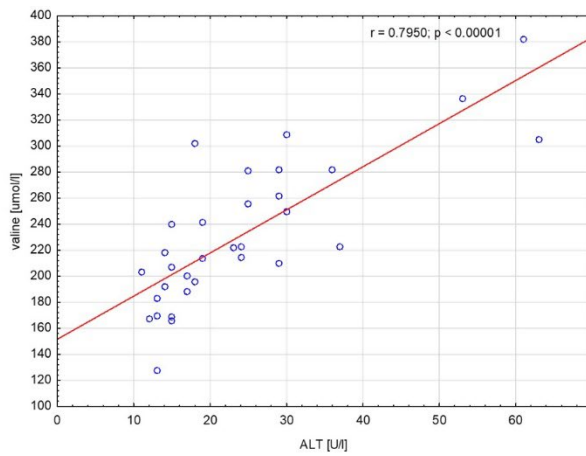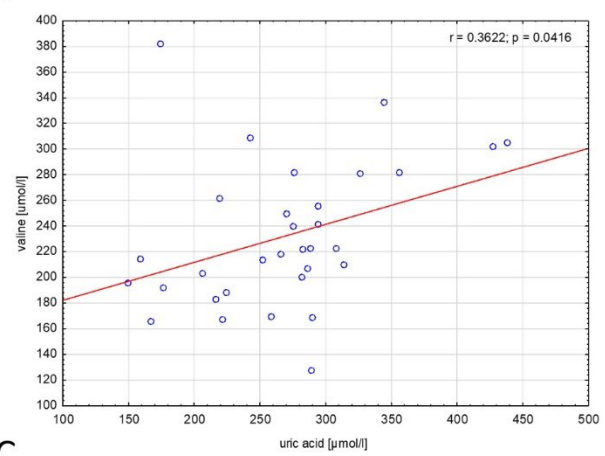

C

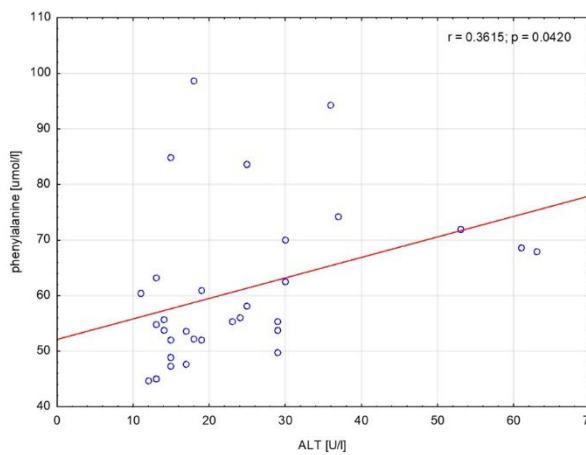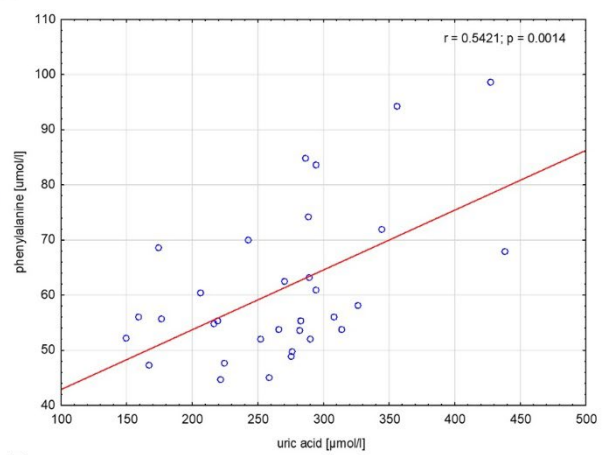

D

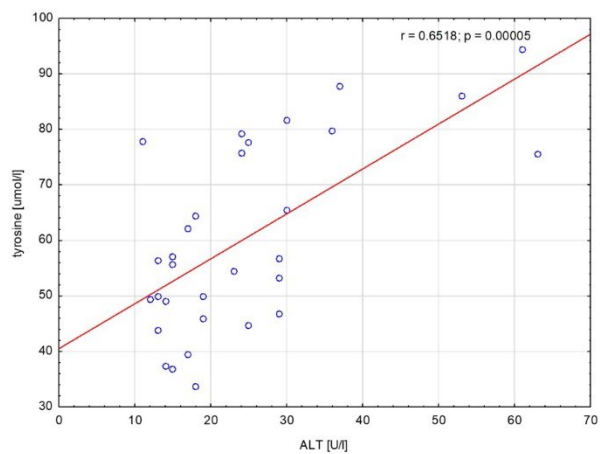

E

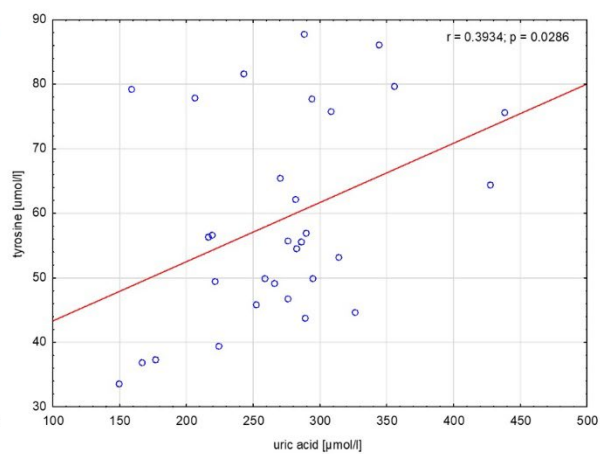

Supplement: SUPPLEMENTARY FIGURE 1 — Correlation between (A) Isoleucine, (B) leucine, (C) valine, (D) phenylalanine, (E) tyrosine and ALT, and uric acid (all children). [file Image_1.pdf]

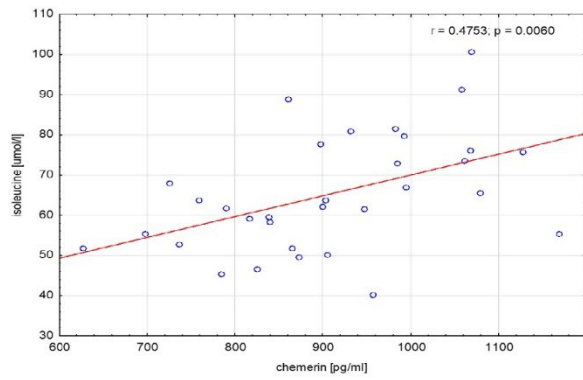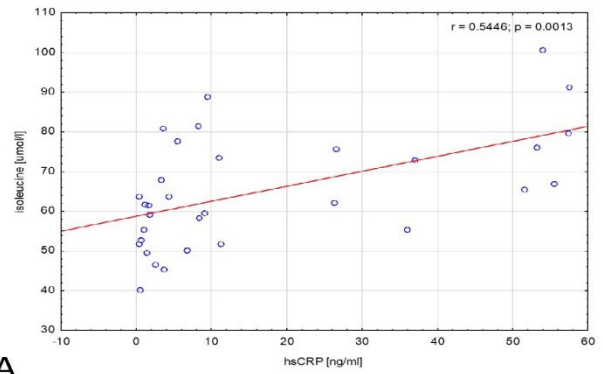

A

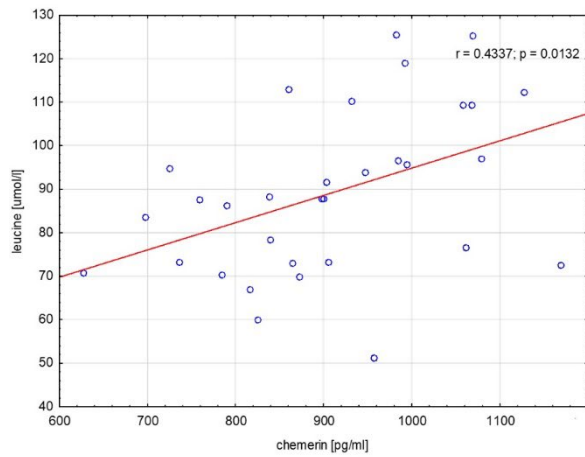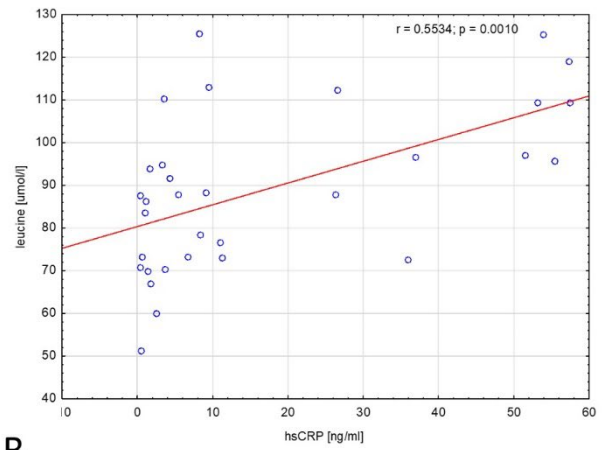

B

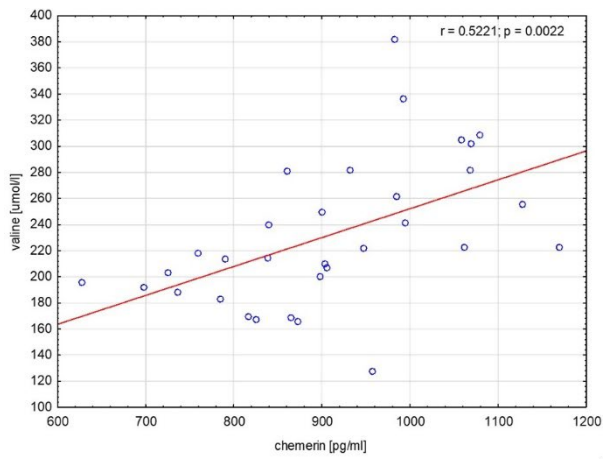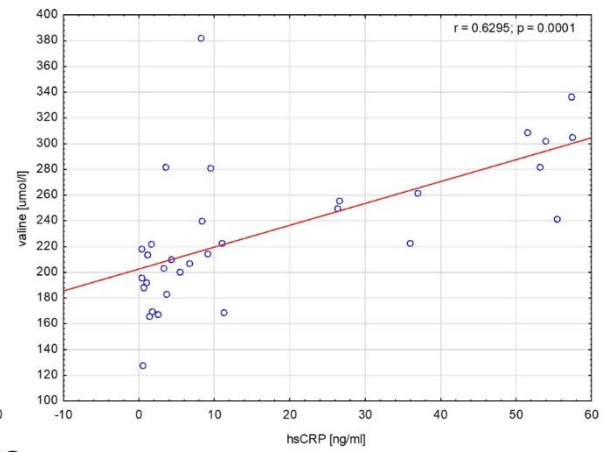

C

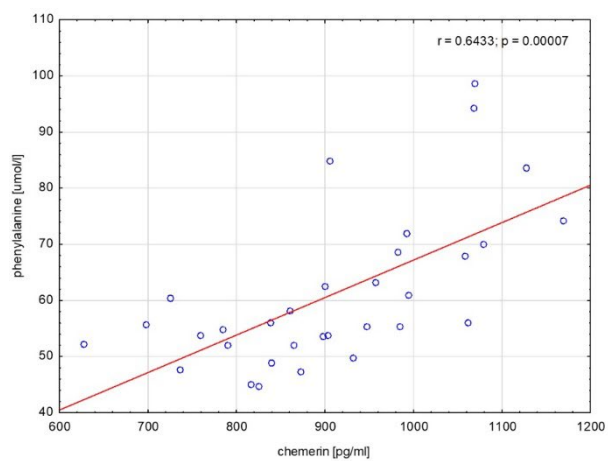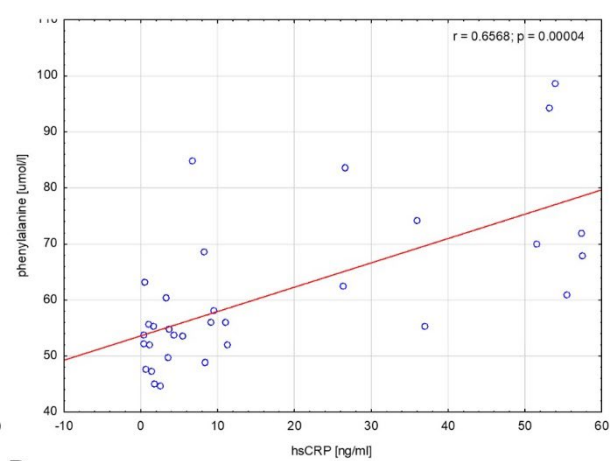

D

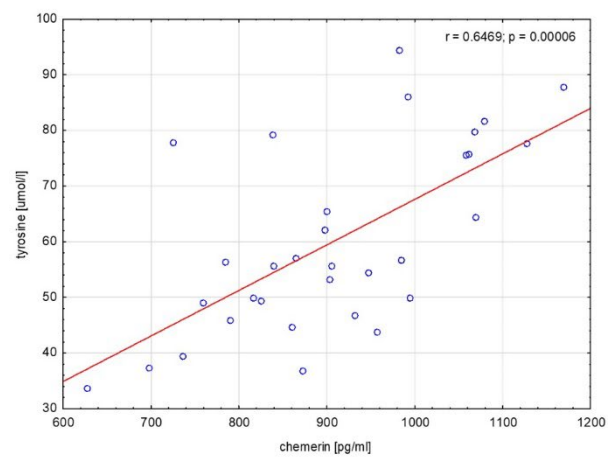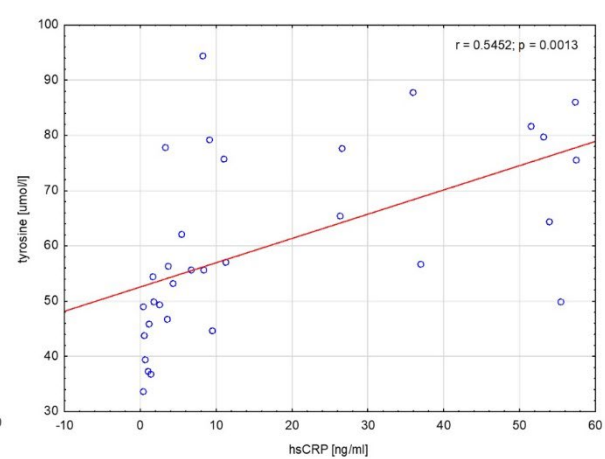

E

Supplement: SUPPLEMENTARY FIGURE 2 — Correlation between (A) Isoleucine, (B) leucine, (C) valine, (D) phenylalanine, (E) tyrosine and hs-CRP, and chemerin (all children). [file Image_2.pdf]
